# Self-Amplifying Pestivirus Replicon RNA Encoding Influenza Virus Nucleoprotein and Hemagglutinin Promote Humoral and Cellular Immune Responses in Pigs

**DOI:** 10.3389/fimmu.2020.622385

**Published:** 2021-01-28

**Authors:** Thomas Démoulins, Nicolas Ruggli, Markus Gerber, Lisa J. Thomann-Harwood, Thomas Ebensen, Kai Schulze, Carlos A. Guzmán, Kenneth C. McCullough

**Affiliations:** ^1^The Institute of Virology and Immunology IVI, Mittelhäusern, Switzerland; ^2^Department of Infectious Diseases and Pathobiology, Vetsuisse Faculty, University of Bern, Bern, Switzerland; ^3^Department of Vaccinology and Applied Microbiology, Helmholtz Centre for Infection Research (HZI), Braunschweig, Germany

**Keywords:** self-amplifying replicon RNA, virus replicon particle, polyplexes, influenza vaccines, humoral and cellular immune response, c-di-AMP adjuvant

## Abstract

Self-amplifying replicon RNA (RepRNA) promotes expansion of mRNA templates encoding genes of interest through their replicative nature, thus providing increased antigen payloads. RepRNA derived from the non-cytopathogenic classical swine fever virus (CSFV) targets monocytes and dendritic cells (DCs), potentially promoting prolonged antigen expression in the DCs, contrasting with cytopathogenic RepRNA. We engineered pestivirus RepRNA constructs encoding influenza virus H5N1 (A/chicken/Yamaguchi/7/2004) nucleoprotein (Rep-NP) or hemagglutinin (Rep-HA). The inherent RNase-sensitivity of RepRNA had to be circumvented to ensure efficient delivery to DCs for intracellular release and RepRNA translation; we have reported how only particular synthetic delivery vehicle formulations are appropriate. The question remained concerning RepRNA packaged in virus replicon particles (VRPs); we have now compared an efficient polyethylenimine (PEI)-based formulation (polyplex) with VRP-delivery as well as naked RepRNA co-administered with the potent bis-(3’,5’)-cyclic dimeric adenosine monophosphate (c-di-AMP) adjuvant. All formulations contained a Rep-HA/Rep-NP mix, to assess the breadth of both humoral and cell-mediated defences against the influenza virus antigens. Assessment employed pigs for their close immunological relationship to humans, and as natural hosts for influenza virus. Animals receiving the VRPs, as well as PEI-delivered RepRNA, displayed strong humoral and cellular responses against both HA and NP, but with VRPs proving to be more efficacious. In contrast, naked RepRNA plus c-di-AMP could induce only low-level immune responses, in one out of five pigs. In conclusion, RepRNA encoding different influenza virus antigens are efficacious for inducing both humoral and cellular immune defences in pigs. Comparisons showed that packaging within VRP remains the most efficacious for delivery leading to induction of immune defences; however, this technology necessitates employment of expensive complementing cell cultures, and VRPs do not target human cells. Therefore, choosing the appropriate synthetic delivery vehicle still offers potential for rapid vaccine design, particularly in the context of the current coronavirus pandemic.

## Introduction

Current influenza vaccines display several disadvantages, such as limited antigen batches, reliance on cell culture or egg production, risk from potential reversion to virulence and interference from pre-existing immunity. Reversion can be circumvented by employing inactivated, split or subunit vaccines, as seen with the majority of current vaccines. However, due to the limited amount of virus antigen present and the absence of self-replication to increase the immunogenic load, the efficacy of these current vaccines is limited. Indeed, they tend to favor induction of humoral defences with poor cell-mediated representation. This relatively poor immunogenicity is also reflected in the need for reformulation for revaccination on an annual basis, due to influenza virus seasonal antigenic drift. In this context, vaccine efficacy can be enhanced by ensuring the promotion of both humoral and cell-mediated defences, as observed with convalescent immunity following virus infection. Replicating vaccines – such as live-attenuated and vector-based vaccines – providing several rounds of antigen production would fulfil such a role (1–4). Yet, live and vector-based vaccines are not without risk.

mRNA-based vaccines are safe and produced under cell-free conditions. In the context of pandemics, they offer potential for rapid vaccine design. Effectively, three candidates are currently in clinical trials to prevent COVID-19: mRNA-1273 (Moderna), BNT-162 (BioNTech), CVnCoV (CureVac). However, a recent study showed that for equivalent levels of protection, self-amplifying (self-replicating) replicon RNA (RepRNA) required a 64-fold lower dose and resulted in prolonged and increased transgene expression (5). RepRNA are more complex and larger molecules than mRNA, encoding a replication machinery and thus resulting in expanded translation of encoded antigens; being derived from defective virus genomes, progeny virus production is excluded (6–14). The replicative nature of RepRNA provides several rounds of antigen production, thus enhancing the antigen dosage available for activating humoral and cell-mediated immunity (CMI), as well as the duration and therefore robustness of that response. These latter points are important in the context of the relative cytopathogenicity of the RepRNA, related to the virus for which it was derived.

It is also important to consider the critical role of dendritic cells (DCs) in immune responses (11, 15, 16). They collect antigen in the periphery, transport it into lymphoid tissues and organs, process the antigen, and promote activation of both humoral and cell-mediated immunity (17–19). In addition to vaccine interaction with DCs, one should also consider the impact of adjuvants selected for vaccine formulation, in terms of the DC maturation essential for efficient immune response induction.

Vaccine interaction with DCs should promote DC functionality in a durable manner, the latter benefiting from a vaccine based on a non-cytopathogenic rather than cytopathogenic virus. Most RepRNA are derived from human pathogens, such as the widely employed alphaviruses. The cytopathogenic nature of alphaviruses can impact on the duration of RepRNA-dependent induction of immune responses. An additional constraint arises from the 5’-cap of alphavirus RNA; recognition of this 5’-cap by cellular innate defences will promote cellular attack against the RepRNA. In contrast, RepRNA derived from classical swine fever virus (CSFV) do not possess these encumbrances. Firstly, they are derived from a non-cytopathogenic porcine pestivirus non-pathogenic for humans. Secondly, they do not carry a 5’-cap, and any viral RNA structure-mediated innate immune activation of the target cells is impeded by the RepRNA-encoded leader autoprotease N^pro^ by a yet unknown mechanism leading to efficient proteasomal degradation of interferon regulatory factor 3 (20).

In order to assess the potential of RepRNA derived from non-cytopathogenic CSFV, we engineered RepRNA to encode influenza virus antigens, including NP or HA from the H5N1 isolate A/chicken/Yamaguchi/7/2004 (H5N1). However, the specific nature of RepRNA requires protection to survive *in vivo* and assistance to cross the cell membrane barrier, particularly to target DCs for internalization (11). Unprotected (“naked”) RepRNA suffer from RNA instability due to particularly high sensitivity to RNase damage of their functionality, and a poor capacity for internalisation into cells. This led to development of virus-like replicon particles (VRPs) (14, 21, 22), or synthetic, nanoparticulate delivery vehicles formulated as chitosan-based particles, polyplexes, or lipoplexes (23–27). Although many of these synthetic formulations were unsuccessful at delivering RepRNA to DCs for translation, particular formulations promote *in vitro* RepRNA delivery and translation, however, still inferior to what is obtained with VRPs.

Of course, RepRNA translation *in vitro* is only of value if also observed *in vivo*. Electroporation of nucleic acids has been reported *in vivo*, but remains painful and does not guarantee DC targeting (28). This leaves the question of how VRP-based delivery would compare with synthetic, nanoparticulate vehicles as an alternative and feasible strategy for delivering RepRNA to DCs. It is also important that the efficacy is assessed in different animal species, especially one with immune responsiveness more closely related to humans. In addition, one can take advantage of potent synthetic adjuvants to enhance further the immune response induction. Accordingly, we assessed RepRNA vaccine delivery by VRP in comparison with a PEI-based polyplex delivery system showing particular efficacy (24). The observations were related back to the *in vitro* characteristics of each delivery system. For *in vivo* assessment, the delivery vehicles carrying the RepRNA were co-administered with the potent adjuvant bis-(3’,5’)-cyclic dimeric adenosine monophosphate (c-di-AMP), monitoring the development of both humoral and cell-mediated immune responses as required for efficacious vaccination against influenza (29–33). This study used a murine model conventionally employed with influenza vaccine pre-clinical evaluation, and a porcine model, due to its closer immunological relationship to humans, in particular in terms of DCs, and being a natural host for influenza virus (34).

## Materials and Methods

### Reagents and Cell Lines

Porcine SK-6 cells (35) were kindly provided by Professor Maurice Pensaert (University of Gent, Belgium). The synthesis and purification of the mucosal adjuvant c-di-AMP was described in Ebensen et al. (30).

### Self-Amplifying Replicon RepRNA

**Figure 1:**
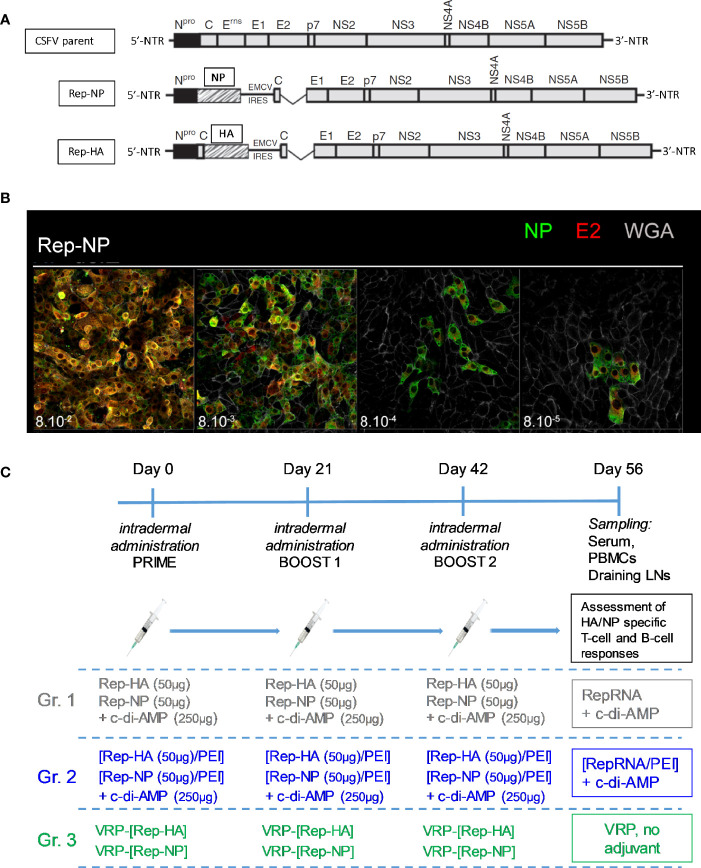
Prime-boost protocol for i.d. pig immunization. **(A)** Schematic representation of the CSFV genome (parent virus genome) employed for generating the RepRNA constructs: the deletion of the E^rns^ coding sequence (ΔE^rns^) and the inserted GOI (Rep-NP, Rep-HA) are shown. **(B)** Illustration of RepRNA functional assay by confocal microscopy, adapted from the infection center assay (ICA) reported elsewhere (25). In this example, SK-6 cells were electroporated with Rep-NP, after what we performed a tenfold serial dilution of the transfected cells (corresponding to 10^-1^ µg RepRNA and 8 x 10^5^ cells per 100 µl in the first dilution to 10^-4^ µg RepRNA and 800 cells per 100 µl in the 4^th^ dilution). 100 µl of each dilution containing electroporated cells were transferred to the corresponding Lab-Tek^®^ wells and let to adhere to 3 x 10^5^ pre-seeded SK-6 cells for 4–6 h in the 37°C incubator in MEM/glutamax/7%v/v-horse-serum. Then, the medium was replaced, and cells were cultured for 48 h at 37°C. The staining protocol involved fixation, permeabilisation and labelling with antibody against NP (green) and E2 (red); cell surfaces were stained with WGA-Alexa633 (gray). **(C)** Illustration of the prime-boost protocol for i.d. pig immunization. On day 56 the peripheral blood and the draining superficial cervical dorsal LNs were collected from sacrificed pigs.

Rep-HA and Rep-NP constructs were already described elsewhere (24–26) and are schematized in **Figure 1A**. They were derived from plasmid pA187-1 that carries a full-length cDNA copy of the genome of the CSFV strain Alfort/187 (CSFV parent) (36) from which the E^rns^ coding sequence was deleted (ΔE^rns^) to engineer the original ΔE^rns^ RepRNA (RepRNA). The Rep-NP was obtained by insertion of the NP gene from influenza virus A/chicken/Yamaguchi/7/2004 (H5N1) (37) *via* a *Not*I restriction endonuclease site at the 3′ end of the N^pro^ coding sequence, upstream of an internal ribosomal entry site (IRES) from encephalomyocarditis virus (EMCV) that permits re-initiation of the translation of the downstream polyprotein. For Rep-HA, a copy of the CSFV C coding sequence was placed between the N^pro^ and the HA glycoprotein coding sequence of the same influenza virus with HA lacking the 5’-terminal 16 codons of the signal peptide. For this, a N^pro^-C-HA DNA cassette was generated by PCR and inserted between the *Cla*I site of N^pro^ and the *Not*I site. This ensures the correct ER translocation of the HA glycoprotein by the E^rns^ signal peptide encoded at the 3-terminal region of C.

### Generation of VRPs

VRPΔE^rns^ carrying a genome with a complete deletion of the E^rns^ coding sequence were produced typically by transfection of 8 x 10^6^ SK-6(E^rns^) cells with 1 μg A187-Δ^Erns^ replicon RNA. The SK-6(E^rns^) cells express the E^rns^ protein required for the generation of VRPΔE^rns^ by trans-complementation (38, 39). The infectious titre of the VRP was determined in SK-6(E^rns^) cells by end-point dilution and expressed in 50% tissue culture infective doses (TCID_50_)/ml according to standard protocols. Based on RT-qPCR quantification of infectious CSFV (of known titre) and of the corresponding full-length genomic *in vitro* transcripts (1µg CSFV genome RNA transcripts = 1.5 x 10^11^ molecules), 1 TCID_50_ of CSFV corresponds approximately to 10^3^ genome equivalents (Hinojosa and Ruggli, unpublished).

### Generation of Synthetic Delivery Vehicles Carrying RepRNA

The generation of chitosan-based nanoparticle delivery formulations, and the PEI-based polyplex nanoparticles were as described previously (23–27).

Briefly, for polyplexes, RepRNA incorporated into polyplex formulations were as follow: [Rep-NP/PEI-4,000 (1:3)] and [Rep-HA/PEI-40,000 (1:2)/(Arg)_9_]. For Rep-NP, RepRNA : PEI-4,000 (weight:weight) ratio of (1:3) was mixed by vortexing (4 s, 10 mM HEPES buffer, pH7.4). After 30 min of incubation at room temperature (RT), volumes were adjusted with serum-free Opti-MEM^®^. For Rep-HA, PEI-40,000 solution was first mixed to 0.5 µM of (Arg)_9_ and further incubated for 30 min at RT. Then, RepRNA was added to the [PEI/(Arg)_9_] core with a RepRNA : PEI (weight:weight) ratio of (1:3), incubate for 30 min at RT. Volumes were adjusted with serum-free Opti-MEM^®^.

### RepRNA Functional Assay Adapted to Confocal Microscopy

All batches of RepRNA production used for the trial were tested by a functional assay adapted to confocal microscopy; the number of cells (expressing CSFV E2) and the gene of interest (GOI) HA or NP were monitored. This assay was described elsewhere (24, 25) and employed reference SK-6 cells that were mixed with RepRNA and electroporated immediately. These cells are very efficient at propagating CSFV and provide a reliable reference to support RepRNA replication (23, 24, 26, 35, 39, 40). SK-6 cell growth is facilitated by Eagle’s Minimal Essential Medium (MEM Earle’s, consisting of MEM supplemented with Earle’s salts, 2 mM l-glutamine, and 7% (v/v) pestivirus- and *Mycoplasma*-free horse serum). The readouts were measured after 48 h; illustrations of E2^+^NP^+^ foci following electroporation of SK-6 cells are shown in **Figure 1B**. The criterion for inclusion of a given RepRNA batch in the trial is detection of E2^+^GOI^+^ foci in the fourth well of a tenfold serial dilution (10^-4^ μg of RepRNA per 8 x 10^2^ SK-6 transfected cells seeded on a pre-formed monolayer of 3 x 10^5^ cells). Ultimately, all the positively-selected RepRNA batches were thawed the day of administration in pigs.

Primary porcine blood DCs (bDCs) were isolated/derived as described (41). Then, they were grown in 8-well fibronectin-coated Lab-Tek^®^ chambers (Nunc, Wiesbaden, Germany). Before exposure to VRP or [RepRNA/PEI], bDCs were washed to remove serum. After 1-3h, cells were washed again and cultured (39°C; DMEM/10%-porcine-serum/50 U/ml-GM-CSF/100 U/ml-IL-4) for 96 h. Labeling used anti-E2 (HC/TC 26, kindly provided by Irene Greiser-Wilke, Hannover, Germany) and anti-NP (HB6J; ATCC, Rockville MD, USA) antibodies. DC-surface staining used WGA-Alexa633 (Molecular Probes/Invitrogen), 10 min on ice, then washed, fixed (4% PFA, 10 min), permeabilized (Perm/Wash-Buffer-I, BD Biosciences, Allschwil, Switzerland). Slides were mounted in Mowiol for confocal microscopy.

### Immunization of Mice and Rabbits

The immunization of mice and rabbits using the chitosan-based nanoparticle (NGA) delivery formulations has been described previously (23). Briefly, RepRNA was incorporated into NGA, and 100 µl were used per dose for vaccination of Balb/c mice and New Zealand white rabbits. Each dose comprised 0.2 µg of Rep-HA in 50 µl plus 0.2 µg of Rep-NP in 50 µl; mice received one dose for each RepRNA mixed together, while rabbits received twice this dose. The NGA vaccines were adjuvanted with 0.5 µg c-di-AMP adjuvant. Animals were vaccinated subcutaneously at days 0, 14, and 28, and bled from the tail vein (mice) or ear (rabbits). Anti-HA and anti-NP titers were assessed by indirect enzyme-linked immunosorbent assay using recombinant HA (H5 Vietnam 2004) and NP as antigen.

The immunization of mice using the polyplex delivery formulations has also been described previously (24, 25). Briefly, C57BL/6 (H-2b) female mice (6–8 week old) were purchased from Harlan (Germany). OVA-TCR transgenic mice C57BL/6-Tg(TcraTcrb)1100Mjb/J (OT-I) were bred at the animal facility of the HZI. All mice were kept under SPF conditions in compliance with the guidelines of the Institutional Animal Use and Care Committee. OT-I (CD8) T cells were sorted from cervical LNs of naive mice (Thy1.1^+^) using Miltenyi CD8 T cell isolation kits. Then, 2.5 × 10^5^ OT-1 CD8 T cells were injected intravenously into recipient C57BL/6 mice, 6–8 week old (Thy1.1^−^) (Day-1). At day 0, mice were vaccinated intrapulmonary with 7.5 μg Rep-OVA and 7.5 μg adjuvant c-di-AMP. Cervical LNs were collected at day 7.

### Immunization of Pigs (Prime-Boost Protocol)

All pigs were obtained from the specific-pathogen-free (SPF) breeding facility of the IVI. Male and female 10-week-old Large White SPF pigs were assigned randomly to three groups of five animals (n=5) housed in separate stables. The experiment was blinded.

Each pig was immunized by intradermal (i.d.) injection of Rep-HA and Rep-NP, co-administered or not with 250 μg of c-di-AMP as adjuvant. During the past few years, we have accumulated data confirming that naked RepRNA is unable to penetrate into DCs for translation, nor does it show evidence for translation *in vivo*. Accordingly, a control group of naked RepRNA alone would have provided no new insights. Instead, groups were constituted as follow: naked RepRNA in association with c-di-AMP (group “RepRNA + c-di-AMP”), reference points for the assay; RepRNA formulated into PEI polyplex delivery vehicles in association with c-di-AMP (group “[RepRNA/PEI] + c-di-AMP”); or RepRNA packaged into VRPs (group “VRP, no adjuvant”). Within each vaccine of the groups “RepRNA + c-di-AMP” and “[RepRNA/PEI] + c-di-AMP”, 50 µg of Rep-HA and 50 µg Rep-NP were applied in a total volume of 0.5 to 0.8 ml each, distributed in five to eight different spots of 0.1 ml each in the dermis of the right and left neck, respectively. For the “VRP” group, 5x10^6^ TCID_50_ of VRP-[Rep-HA] and 5x10^6^ TCID_50_ of VRP-[Rep-NP] were applied in a total volume of 0.5 to 0.8 ml each, distributed in five to eight different spots of 0.1 ml each in the dermis of the right and left neck, respectively. 5x10^6^ TCID_50_ of VRP corresponds approximately to 5x10^9^ viral genomes (see above), which represents a weight of 33 ng of RNA (1µg CSFV genome = 1.5x10^11^ genome molecules, see above). Thus, compared with VRP, approximately 10^3^ times more RepRNA (50 µg) was applied with the PEI polyplex delivery system.

A total of three immunizations were applied with 3 weeks interval. At day 56, animals were euthanized by electrical stunning and subsequent exsanguination, blood was taken and superficial cervical dorsal lymph nodes (LNs) were collected (**Figure 1C**). Importantly, naked RepRNA co-administered with c-di-AMP, the VRPs and the PEI-complexes were well tolerated by the animals, and no side effects were observed over the 56-day observation period (data not shown).

### HA and NP Restimulation of Freshly Isolated Cells

PBMC were isolated by centrifugation on a Ficoll-paque density gradient (1.077 g/L, GE Healthcare. Chicago, IL, USA). Harvested LNs were washed with sterile PBS^+/+^, cut in small pieces, and incubated in 15 ml of pre-warmed PBS^+/+^ containing 0.36 mg/ml collagenase D (Sigma-Aldrich) and 100 µg/ml DNase I (Sigma-Aldrich) during 30 min under agitation at 37°C. The enzymatic reaction was stopped by adding 50 ml of PBS^-/-^ with 5 mM EDTA solution. The cells were then filtered through a 100 µm and 70 µm filter (BD Biosciences, Allschwil, Switzerland), washed three times with PBS^-/-^ (4°C). Cell count and viability was done with Türk’s solution under the microscope.

Isolated PBMCs or LN cells for T cell and B cell restimulation assays (1 x 10^6^ cells/ml) were cultured in DMEM/10%FBS with 1 µg/ml recombinant HA/NP (Immune Technology Corp., New York, U.S.A.), 1 µg/ml recombinant E2 [produced in cultures of insect cells infected with the baculovirus vector (42)], or without (unstimulated). As a positive control, the cells from all samples were cultured with 10 µg/ml of Concanavalin A (Con A) (Sigma). Importantly, those experiments were performed in the absence of antibiotics. After 3 or 5 days at 39°C, cells were harvested and analyzed by flow cytometry (FCM).

### Flow Cytometry

FCM combination staining for T cell and B cell proliferation by CellTrace™ Violet (Life Technology) dilution employed: CD3 (BB23-8E6-8C8) (BD Biosciences), CD4 (PT90A), CD8 (76-2-11), CD21 (BB6.11C9.6), and IgM (PIG 45A) (all from VMRD, Germany). 5x10^5^ events were stained for each sample and the whole tube was acquired. Analysis gate was placed on FSC^high^ cells, as proliferating T cells are located at this area (24). Proliferating CellTrace^low^ cells were scored as a percentage of the FSC^high^ cell gate. Data were acquired using FACSCanto II (Becton-Dickenson), and analyzed with Flowjo software (version 9, Treestar, USA).

### Serum Antibodies

Anti-HA and anti-NP antibody titers were assessed by indirect ELISA as described previously (23).

### Statistical Analysis

Statistical analysis was done using the GraphPad Prism 8 software (GraphPad software, La Jolla, CA, USA). The *p*-values were calculated using one-way ANOVA and Bonferroni nonparametric post-test. Data are presented as box and whisker plots (* p <0.05, ** p <0.01, *** p <0.001).

## Results

### Delivery of RepRNA in Rabbits and Mice

**Figure 2:**
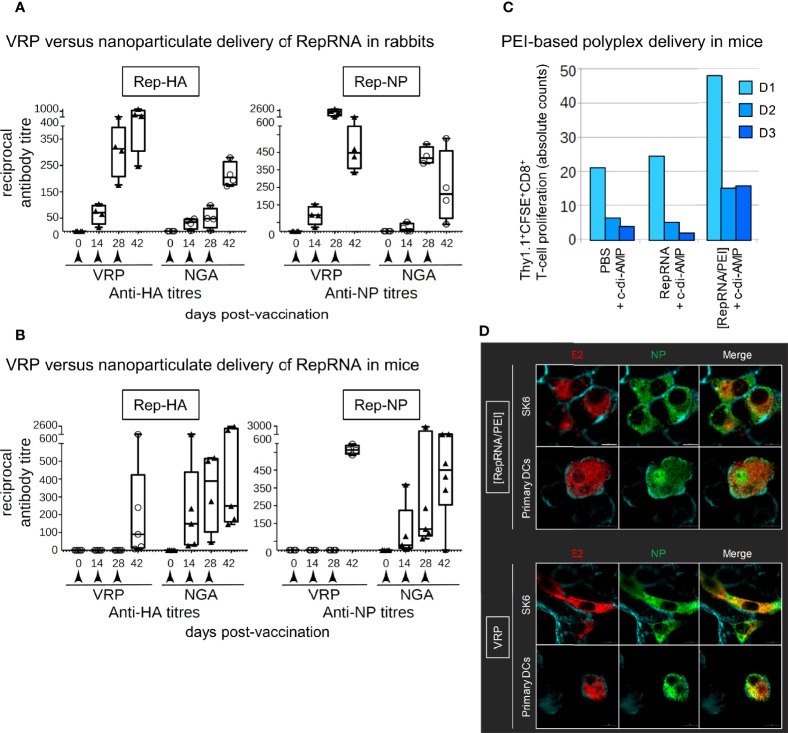
Virus replicon particle (VRP) and NGA deliver RepRNA for translation *in vivo*. **(A, B)** Mice and rabbits were injected subcutaneously at 0, 14, and 28 days. Rep-HA and Rep-NP were delivered in NGA or VRP; all vaccines were adjuvanted with c-di-AMP adjuvant. Serum samples were assessed for anti-HA and anti-NP antibodies by ELISA and titers estimated at the times shown. **(C)** Adoptive transfer studies in mice have been performed in order to investigate the potential of the polyethylenimine (PEI) formulations to stimulate cellular immune responses. In brief, 24 h prior vaccination of wild type mice, CFSE labeled Thy1.1 CD8^+^ T cells of antigen-specific TCR transgenic mice were injected intravenously. 7 days later, cervical LNs of transplanted mice were collected and the proliferative capacity of the CFSE labeled Thy1.1 CD8^+^ T cells was analyzed by flow cytometry. Cell number as well as the number of cell divisions correlate with both strength of the stimulated cellular response and vaccine delivery efficacy. The number of division cycles (D1-D3) of proliferating OT-I CD8^+^ T cells were determined by flow cytometry (FCM) using the FlowJo software. For each group, tissues from five mice were pooled to provide enough cells. **(D)** Translation of delivered Rep-NP in porcine SK-6 cells and primary dendritic cells (DCs). After incubation for 48 h at 37°C with Rep-NP complexed to PEI or packaged into VRP, the cells were washed, fixed (4% PFA), permeabilised (saponin) and labeled with antibodies against influenza NP (green) and CSFV E2 (red); cell surfaces were stained with WGA (blue). All pictures were generated with IMARIS 7.7 with threshold subtraction and gamma correction set as in the “RepRNA alone” control.

Comparison of the previously reported chitosan-alginate nanoparticulate (NGA) delivery with VRPs using RepRNA encoding influenza virus HA and NP showed a clear advantage for VRP delivery in rabbits (**Figure 2A**). In contrast, the NGA delivery appeared to be the more advantageous in mice, in terms of an early induction of specific immunity; the VRPs required three immunizations before a detectable specific antibody response was obtained (**Figure 2B**). Moreover, VRP efficiency in mice was variable between experiments, with little or no specific antibody induced in certain cases (data not shown).

The readouts obtained with the NGA in mice also showed variation between experiments, as did the use of lipids (27) or polyplexes (24, 26) for delivery. It was discovered that the efficiency of polyplex delivery leading to translation of the RepRNA was highly dependent on the PEI formulation (24), and it appears that the potential for lipoplexes suffers from the same encumbrance (27). Finally, we considered the efficiency of polyplex delivery formulations to induce cellular immune response. Antigen-specific proliferation of OT-I CD8^+^ T cells were assessed 7-days post-immunisation in cells collected from cervical LNs. Reduced CFSE signal from preloaded cells (due to cell division distributing the dye to daughter cells) was employed as readout. In case of the CD8^+^ T-cell compartment, immunisation with polyplex was efficient to induce CD8^+^ proliferative responses compared to PBS and naked RepRNA treated mice. These *in vivo* results demonstrate that polyplexes combined with the mucosal adjuvant c-di-AMP promotes translation of encoded antigens by DCs to stimulate proliferation of GOI-specific CD8^+^ T cells (**Figure 2C**), making important to assess their efficiency in the porcine model, due to the closer immunological relationship to humans.

### Assessment of Delivery to DCs *In Vitro*

Before proceeding with the assessment of RepRNA delivery in pigs, it was considered important to ascertain the efficiency with which the polyplexes and VRPs delivered RepRNA for translation of the inserted influenza virus genes. This was initially determined using porcine SK-6 cells and blood DCs, relating to our previous publications for chitosan-based, lipid-based, and polyplex-based delivery of the RepRNA (6, 23–27). **Figure 2D** shows that both the polyplexes and VRPs were able to deliver RepRNA leading to translation of the gene encoding influenza virus NP antigen.

### VRP Immunization of Pigs Induces Antigen-Specific Cells in Draining Lymph Nodes

**Figure 3:**
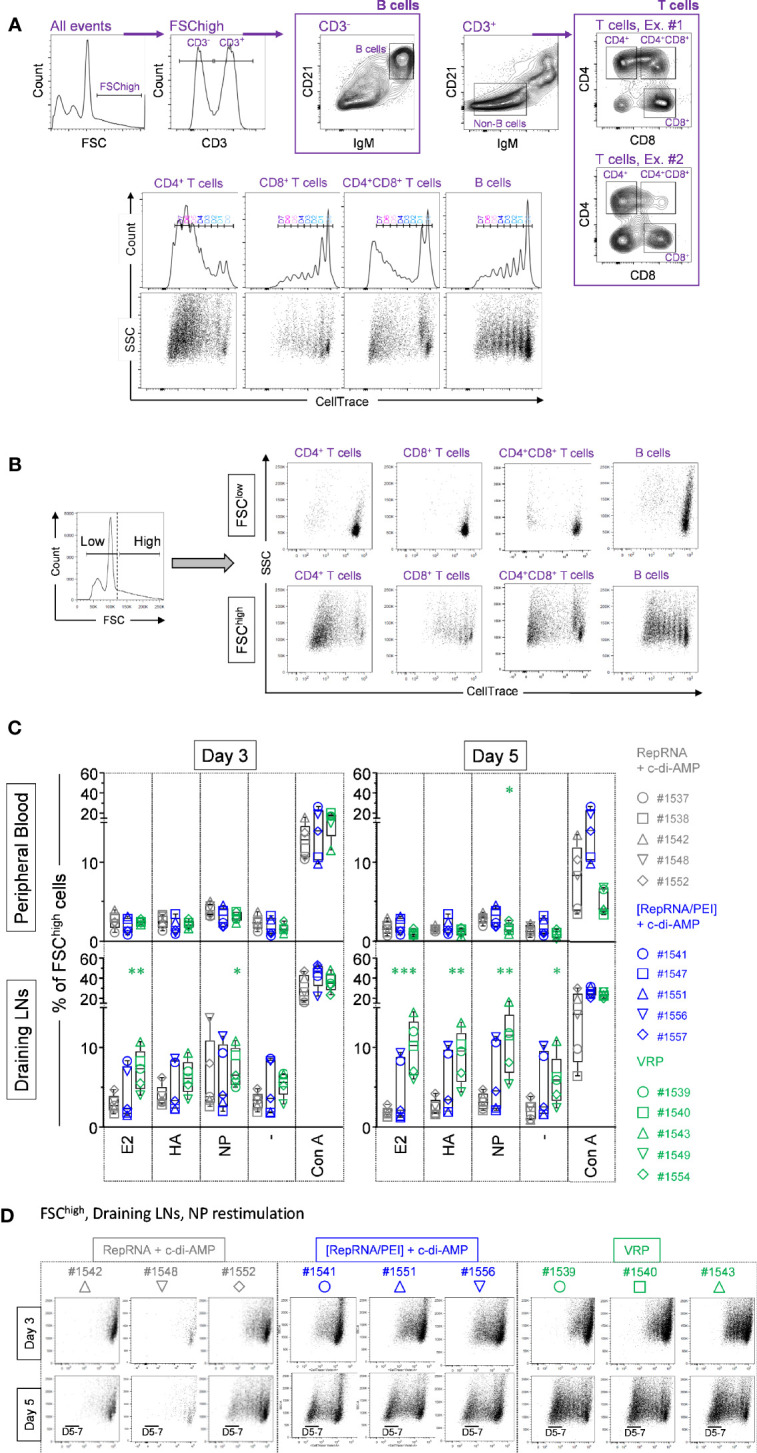
Virus replicon particle (VRP) immunization induces specific immune cell proliferation in the draining lymph nodes. **(A)** The gating strategy used to quantify B and T cell proliferation, based on the expression of CD3, CD4, CD8, CD21, IgM, and CellTrace™ markers, is shown. **(B)** On the left side is shown a representative histogram, where FCS^low^ and FSC^high^ gates can be distinguished. Then, CD4^+^ T cells (CD3^+^CD21^-^IgM^-^CD4^+^CD8^-^), CD4^+^CD8^+^ T cells (CD3^+^CD21^-^IgM^-^CD4^+^CD8^+^), CD8^+^ T cells (CD3^+^CD21^-^IgM^-^CD4^-^CD8^+^), and B cells (CD3^-^CD21^+^IgM^+^CD4^-^CD8^-^) where analyzed in parallel from those two independent FSC^low^ and FSC^high^ gates. The representative examples on the right side clearly show that proliferative cells are CellTrace^low^ FSC^high^. **(C, D)** At day 56 post-first immunization, freshly isolated PBMCs and LN cells where exposed against recombinant CSFV E2 and influenza virus HA and NP, or were let unstimulated (“-”). For all samples, Con A was used as a positive control. **(C)** Induction of FSC^high^ cell population following Ag restimulation. Cells from the individual animals are represented by separate symbols. The percentage of FSC^high^ cells was determined by flow cytometry with FlowJo. (*p ≤ 0.05; **p ≤ 0.01; ***p ≤ 0.001). **(D)** After 3 or 5 days of cell culture, the numbers of division cycles using CellTrace™ of proliferating cells from FSC^high^ subset from restimulated LN cells were determined by flow cytometry with FlowJo. Representative animals are shown for the three animal groups “RepRNA + c-di-AMP”, “[RepRNA/PEI] + c-di-AMP” and “VRP”.

Next, we compared polyplex- and VRP-mediated delivery of RepRNA in pigs with the immunization scheme shown in **Figure 1C**. Cells collected from peripheral blood and draining LNs of immunized pigs were restimulated *in vitro* with CSFV E2, or influenza virus HA/NP recombinant proteins. First, we quantified the percentage of FSC^high^ cells for all unstimulated and restimulated samples, since porcine proliferating lymphocytes exhibit forward scatter of a higher intensity (24) (example shown in **Figures 3A, B**).

Lymphocyte activity in the peripheral blood was assessed using PBMCs derived from comparators immunized with “RepRNA + c-di-AMP”. Polyclonal restimulation of PBMCs *in vitro* employed Con A to ascertain the responsiveness of the cells. A high and comparable percentage of FSC^high^ cells was observed with cells from animals in the vaccination groups, particularly at day 3 (**Figure 3C**, upper panel, Con A). This indicated that the PBMCs retained a good and comparable intrinsic capacity for proliferation, whether they had been exposed *in vivo* to “RepRNA + c-di-AMP”, “[RepRNA/PEI] + c-di-AMP” or “VRP”. When antigen-specific stimulation of the PBMC was assessed with E2, HA or NP antigens, PBMCs derived from pigs receiving “[RepRNA/PEI] + c-di-AMP” or “VRP” failed to display a clear enriched FSC^high^ population in peripheral blood on day 3 and 5 (**Figure 3C**, upper panel).

Lymphocytes were also derived from draining LNs. Their responsiveness to Con A was similar to that observed in PBMCs (**Figure 3C**, lower panel), implying a preserved capacity to proliferate for all animals in the study. In contrast to PBMCs, a more notable antigen-specific stimulation was observed, particularly when using NP antigen. Comparators immunized with “RepRNA + c-di-AMP” showed low evidence for specific cell proliferation (low percentage of FSC^high^ cells when resimulated *in vitro* with E2 and HA at day 3 and 5, whereas an enhanced proliferation of cells was observed in two of five pigs following NP restimulation at day 3) (**Figure 3C**, left lower panel). No significant improvement was seen for the group “[RepRNA/PEI] + c-di-AMP”. In contrast, cells derived from draining LNs of pigs immunized with VRP gave a clear enriched FSC^high^ population, most notable at day 5 (E2 (***), HA (**) and NP (**)). Altogether, these results show a clear distinction between pigs immunized with naked RepRNA or polyplexes (weak FSC^high^ cell enrichment at day 3) and pigs immunized with VRPs (specific FSC^high^ cell enrichment occurring in the draining LNs at days 3-5 p.i.). This increased FSC^high^ cell population strongly suggested a specific T- and B-cell response against influenza virus antigens.

### Confirmation of Antigen-Specific Proliferation With Draining Lymph Node Cells From Polyplex and VRP Immunized Pigs

In order to confirm the above results, we assessed cell proliferation by reduction in the CellTrace signal from preloaded cells (due to cell division distributing the dye to daughter cells). **Figure 3D** displays examples of the dot plots gated on FSC^high^ cells from draining LNs, following NP restimulation. Cells from comparators receiving “RepRNA + c-di-AMP” lacked clear evidence for specific proliferation – the percentage of CellTrace^low^ was low, remaining stable (pig #1542) or only slightly increased (pigs #1548 and #1552) between days 3 and 5. In contrast, cells from animals receiving “[RepRNA/PEI] + c-di-AMP” showed proliferation – the percentage of CellTrace^low^ increased between day 3 and 5. Cells from animals immunised with VRP showed a higher proliferation – the percentage of CellTrace^low^ was high, with strong increases between day 3 and 5. Altogether, these results established that RepRNA must be correctly packaged, either in PEI-based polyplex or in VRPs, for efficient delivery *in vivo* leading to intracellular release of the RepRNA for translation of the encoded antigens to promote specific cell proliferation in secondary lymphoid organs. Of note, VRP were clearly more efficient at genome delivery than the PEI-based polyplexes since the latter required 10^3^ times more RNA than the VRP for comparable immune induction (see materials and methods).

### Polyplex and VRP Induce CD4^+^ T Cell Responses Specific for Encoded Influenza Virus Antigens

**Figure 4:**
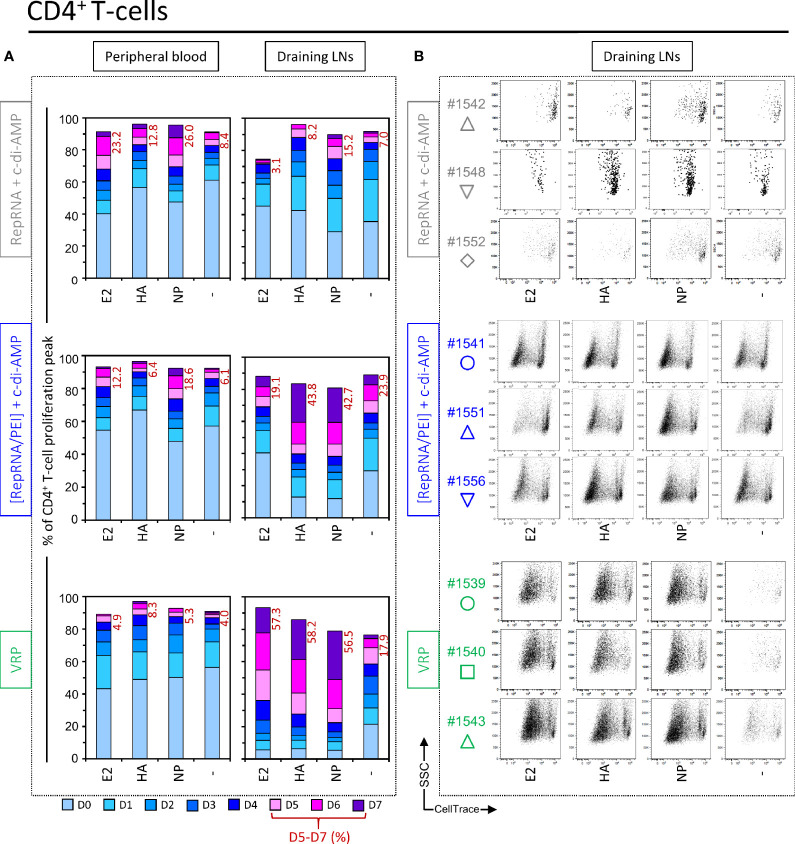
Virus replicon particle (VRP) and polyplex induce a strong and specific CD4^+^ T cell immune response against influenza antigens. Induction of CD4^+^ T cell immune responses against the RepRNA encoded CSFV E2 and influenza virus HA and NP, determined by restimulation of freshly isolated PBMCs and LN cells (day 56 post-first immunization); cells were restimulated with recombinant E2 (1 µg/ml), HA (1 µg/ml) or NP (1 µg/ml), or non-stimulated (-). **(A)** After 5 days of cell culture, the numbers of division cycles (D0-D7) of proliferating CD4^+^ T cells in PBMC and draining LN compartments, were determined by flow cytometry using the FlowJo software. The values represent the mean of proliferating CD4^+^ T cells of the five individual pigs for each group. Numbers in red represent the mean percentage of highly proliferative cells (≥ 5 division cycles). **(B)** After 5 days of cell culture, representative dot plots are shown for draining LN compartments of three immunized animals per groups for proliferating CD4^+^ T cells.

Considering the apparent advantage of VRP delivery leading to RepRNA translation of the encoded influenza virus antigens inducing specific immune responses, the question arose as to the compartmentalisation of these responses. Firstly, CD4^+^ T cell proliferation was measured using *in vitro* restimulation with E2, HA, and NP recombinant proteins, using again the CellTrace signal from preloaded cells as readout. By day 5, a moderate proliferation of CD4^+^ T cells was observed in unstimulated wells from certain comparators, up to seven rounds of cell division. This background proliferation was slightly increased after *in vitro* restimulation with NP. In comparison, peripheral blood cells from pigs receiving polyplexes or VRPs also showed a slight background proliferation of CD4^+^ T cells in unstimulated wells. As with the comparators, this proliferation increased in response to influenza virus NP, more substantially for “[RepRNA/PEI] + c-di-AMP” group (**Figure 4A**, Peripheral blood).

The above results with LN cells from the “RepRNA + c-di-AMP” group proved to be relatively minor compared to results obtained with cells from pigs receiving polyplexes and VRPs. Notably for the latter, CD4^+^ T cells displayed a very strong proliferation in response to the E2, HA and NP antigens. This was observed as a very high percentage of division peaks D5 – D7 for E2 (57.3%), HA (58.2%), and NP (56.5%), compared to background level for the unstimulated “-” (17.9%) (**Figure 4A**, Draining LNs). Dot plots for these results are shown in **Figure 4B** for three animals from each group. Altogether, these results demonstrate that immunization with RepRNA packaged in PEI-based polyplex or VRP promotes translation of encoded antigens, likely by DCs, in pigs.

### Polyplex and VRP Induce CD8^+^ T Cell Responses Specific for Encoded Influenza Virus Antigens

**Figure 5:**
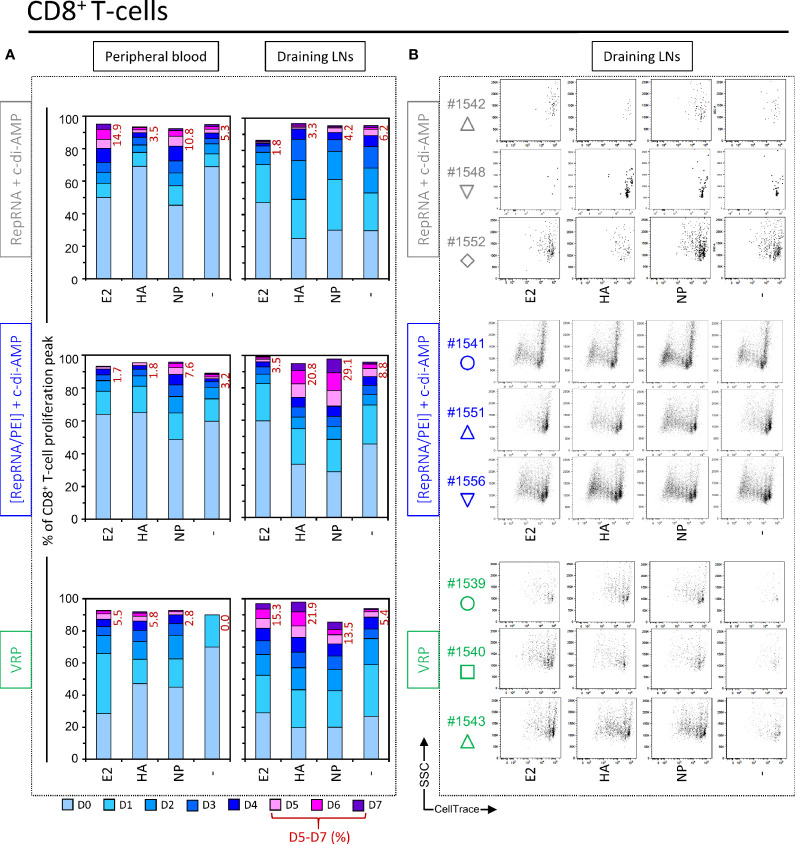
Virus replicon particle (VRP) and polyplex induce a specific CD8^+^ T cell immune response against influenza antigens. Induction of CD8^+^ T cell immune responses against the RepRNA encoded CSFV E2 and influenza virus HA and NP, determined by restimulation of freshly isolated PBMCs and cells derived from draining LNs (day 56 post-first immunization); cells were restimulated with recombinant E2 (1 µg/ml), HA (1 µg/ml) or NP (1 µg/ml), or non-stimulated (-). **(A)** After 5 days of cell culture, the numbers of division cycles (D0-D7) of proliferating CD8^+^ T cells in PBMC and draining LN compartments, were determined by flow cytometry using the FlowJo software. The values represent the mean of proliferating CD8^+^ T cells of the five individual pigs for each group. Numbers in red represent the mean percentage of highly proliferative cells (≥ 5 division cycles). **(B)** After 5 days of cell culture, representative dot plots are shown for draining LN compartments of three immunized animals per group for proliferating CD8^+^ T cells.

In addition to the above assessment of CD4^+^ T cell responses, the same analysis was applied to cytotoxic CD8^+^ T cells. Again, PBMCs and cells isolated from draining LNs of most pigs immunized with “RepRNA + c-di-AMP” failed to display a sufficient FSC^high^ population for accurate analysis (only one out of five pigs). Restimulation of its CD8^+^ T cells with E2 and NP induced a weak cell proliferation with peaks D5 – D7 for E2 (14.9%) and for NP (10.8%) compared to background, unstimulated levels (“-”, 5.3%) (**Figure 5A**, top panel, “Peripheral blood”). However, cells derived from draining LNs failed to induce significant anti-E2, anti-HA or anti-NP response, when compared to background proliferation in unstimulated wells (“-”.) (**Figure 5A**, top panel “Draining LNs” and **Figure 5B**). This supports the notion that specific proliferation in response to the encoded influenza virus antigens was at best moderate for the unique responder animal in this vaccination group.

Like the above comparator animals, no clear anti-E2, anti-HA, or anti-NP CD8^+^ T cell response responses were detectable in peripheral blood samples of pigs immunized with “[RepRNA/PEI] + c-di-AMP” (**Figure 5A**, middle panel, “Peripheral blood”). However, cells derived from draining LNs showed clear CD8^+^ immune responses against influenza antigens, and particularly for NP (increased frequency at day 5 of peaks D5 – D7 (**Figure 5A**, middle panel, “Draining LNs”). This is also illustrated in the dot plots shown in **Figure 5B**. Peaks of low CellTrace intensity were easily detected, even if at lower percentage than those measured with CD4^+^ T cells.

Also for pigs immunized with “VRP”, no clear anti-E2, anti-HA or anti-NP responses were detectable in peripheral blood samples (**Figure 5A**, bottom panel, “Peripheral blood”). Again, cells derived from draining LNs showed clear CD8^+^ immune responses against influenza antigens, albeit at lower percentage for NP than those measured in pigs immunized with polyplexes (**Figure 5A**, “Draining LNs” and **Figure 5B**). In conclusion, the combined observations for CD4^+^ and CD8^+^ T cells in pigs receiving RepRNA packaged in PEI-based polyplexes or VRPs, confirmed that the T-lymphocytes had clearly responded to RepRNA encoded GOI (HA and NP antigens), which could only have arisen following RepRNA translation *in vivo*.

### VRP and Polyplex Activate CD4^+^CD8^+^ T Cells Response Against Influenza Antigens

**Figure 6:**
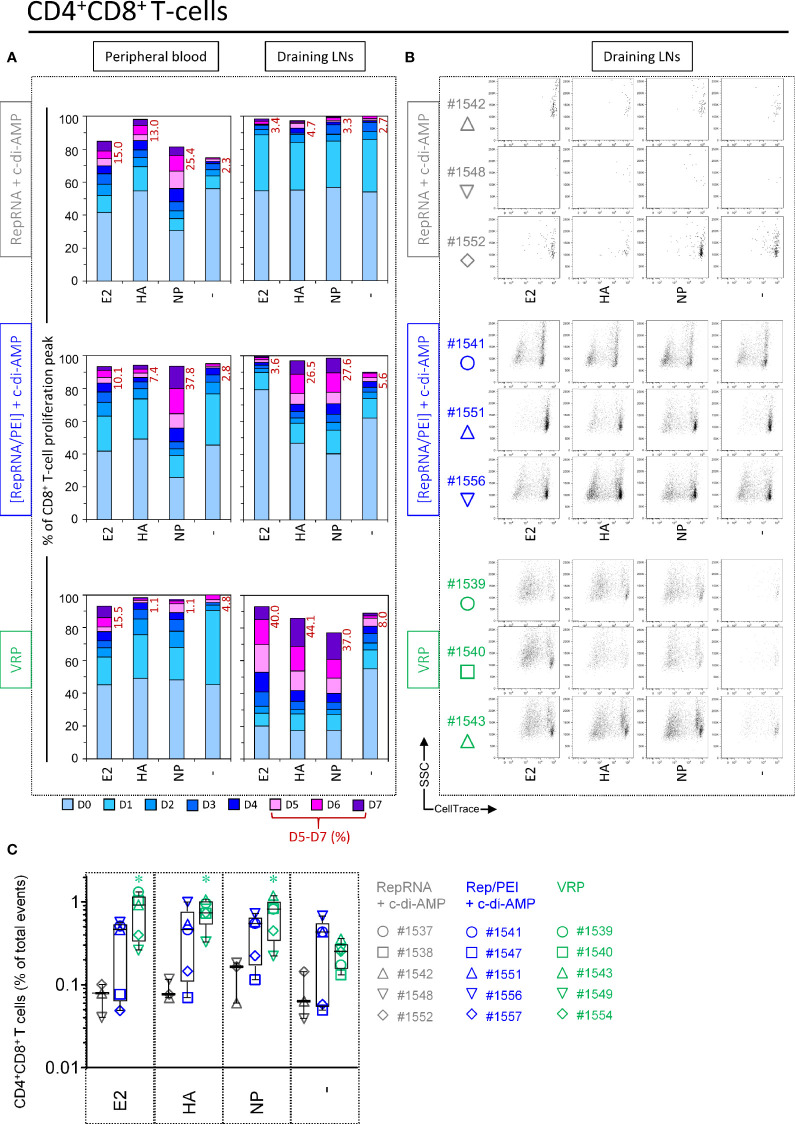
Virus replicon particle (VRP) and polyplex activate CD4^+^CD8^+^ T cells response against influenza antigens. Induction of CD4^+^CD8^+^ T cell immune responses against the RepRNA encoded CSFV E2 and influenza virus HA and NP, determined by restimulation of freshly isolated PBMCs and cells derived from draining LNs (day 56 post-first immunization); cells were restimulated with recombinant E2 (1 µg/ml), HA (1 µg/ml) or NP (1 µg/ml), or non-stimulated (-). **(A)** After 5 days of cell culture, the numbers of division cycles (D0-D7) of proliferating CD4^+^ T cells in PBMC and draining LN compartments, were determined by flow cytometry using the FlowJo software. The values represent the mean of proliferating CD4^+^CD8^+^ T cells of the five individual pigs for each group. Numbers in red represent the mean percentage of highly proliferative cells (≥ 5 division cycles). **(B)** After 5 days of cell culture, representative dot plots are shown for draining LN compartments of three immunized animals per group for proliferating CD4^+^CD8^+^ T cells. **(C)** For the counts of CD4^+^CD8^+^ T cell subset, we calculated the percentage of total events: ratio (number of events in the gated cell subtype: FSC^high^CD3^+^CD21^-^IgM^-^CD4^+^CD8^+^) to (number of all events). *p ≤ 0.05.

CD4^+^CD8^+^ double-positive (DP) lymphocytes proliferate in response to stimulation with recall viral antigen and include most likely memory/effector T cells [for a review see (43)]. For PBMCs, a moderate proliferation of DP T cells was observed in unstimulated wells from certain comparators. This background proliferation was slightly increased after NP restimulation. However, this clear anti-NP response was higher in pigs receiving polyplexes (peaks D5–7 = 37.8%), whereas no clear response were detectable in peripheral blood samples from pigs immunized with “VRP (**Figure 6A**, Peripheral blood).

Once again, the above results proved to be relatively minor compared to what observed with cells isolated from draining LNs. DP T cells derived from pigs receiving polyplexes showed clear DP T cell immune responses against influenza antigens (**Figure 6B**). But this was moderate compared to pigs immunized with VRPs, where DP T cells displayed a very strong proliferation in response to E2, HA and NP (percentage of division peaks D5 – D7: 40.0%, 44.1%, and 37.0%, respectively) (**Figure 6A**, Draining LNs). This is also illustrated in the dot plots shown in **Figure 6B**.

Given that DP T cells displayed strong proliferations in LN samples following restimulation, we next wondered whether the number of this specific subset could increase in animals receiving either polyplexes or VRPs. Expansion could be indicative of the induction of a memory T cell response (as shown in **Figure 3A**, large differences could be seen in the frequency of this T cell subset depending of the experimental condition). Effectively, a significant increase in size was observed following antigen restimulation for pigs receiving VRPs, whereas three out of five pigs receiving polyplexes displayed a clear enrichment of DP T cells (**Figure 6C**). Taken together, these results strongly suggest that our vaccine formulations can induce an immunological T cell memory against influenza antigens.

### VRP Induce a Specific B Cell Immune Response Against Influenza Virus Antigens

**Figure 7:**
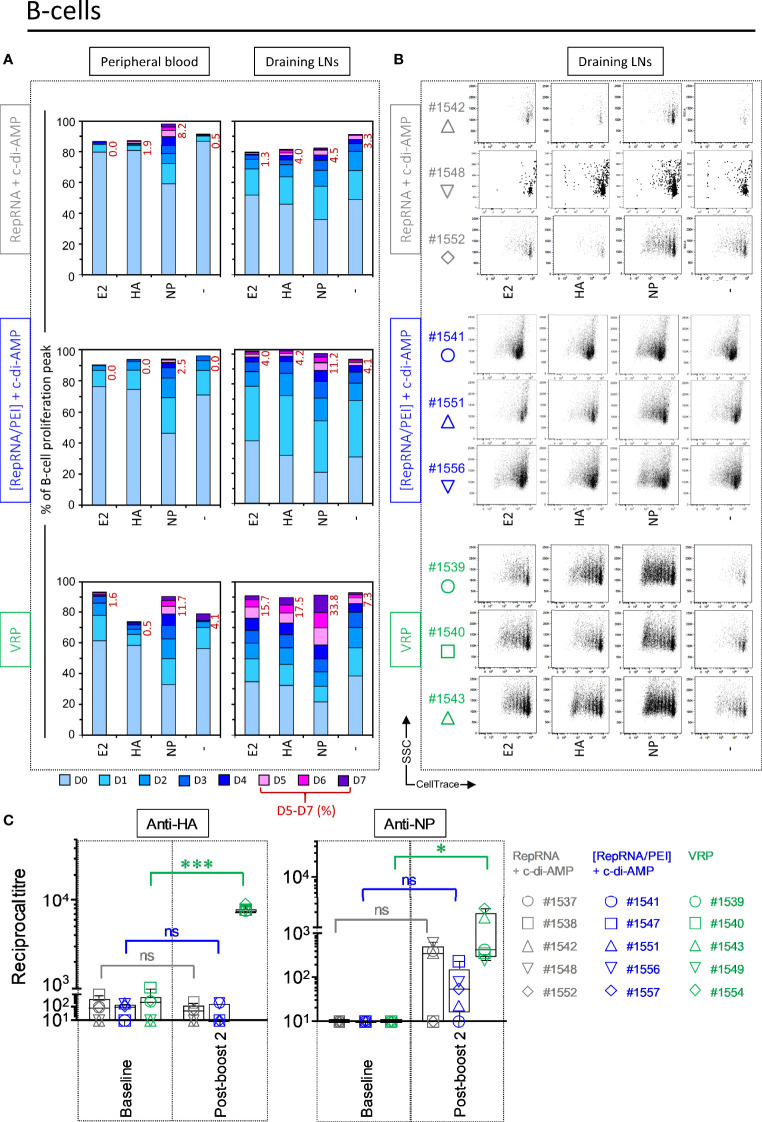
Virus replicon particle (VRP) induce a strong and specific humoral immune response against influenza antigens. Induction of B cell immune responses against the RepRNA encoded CSFV E2 and influenza virus HA and NP, determined by restimulation of freshly isolated PBMCs and cells derived from draining LNs (day 56 post-first immunization); cells were restimulated with recombinant E2 (1 µg/ml), HA (1 µg/ml) or NP (1 µg/ml), or non-stimulated (-). **(A)** After 5 days of cell culture, the numbers of division cycles (D0-D7) of proliferating B cells in PBMC and draining LN compartments, were determined by flow cytometry using the FlowJo software. The values represent the mean of proliferating B cells of the five individual pigs for each group. Numbers in red represent the mean percentage of highly proliferative cells (≥ 5 division cycles). **(B)** After 5 days of cell culture, representative dot plots are shown for immunized animals per group for proliferating B cells. **(C)** Induction of humoral immune response (IgG titer) against RepRNA encoded influenza virus HA and NP, determined by ELISA using day -5 (baseline) and day 56 (post-boost 2) sera from individual pigs. ***p < 0.001; *p < 0.05.

Considering the importance of the humoral response against influenza virus, specific B cell response against the RepRNA encoded influenza virus antigens was assessed. For PBMCs derived from pigs immunized with “RepRNA + c-di-AMP” that displayed FSC^high^ cells, a clear anti-NP response was observed (at day 5, peaks D5-7 = 8.2%). However, this specific anti-NP response was higher in pigs receiving the antigens using VRPs (peaks D5-7 = 11.7%) (**Figure 7A** “Peripheral blood”).

B cells derived from the LNs of comparator pigs showed no sign of activation of E2-, NP- and HA-specific B cells, respectively. Those readouts were also negligible in pigs receiving polyplexes, with the slight exception of NP restimulation. In contrast, very strong B cell proliferation was observed in pigs receiving VRPs in response to E2, HA and particularly NP (**Figure 7A**, “Draining LNs”). **Figure 7B** recapitulates the percentages of CellTrace^low^ division peaks D5 – D7 for E2 (15.7% ± 0.8), HA (17.5% ± 4.2), and NP (33.8% ± 1.5), compared to background, unstimulated levels (“-”, 7.3% ± 4.0) in pigs receiving polyplexes and VRPs.

### VRP Induced Lymphocyte Responses Translate to Antibodies Against Influenza Virus Antigens

The above results clearly demonstrate that the VRP-delivered RepRNA, and to a lesser extent PEI-delivered RepRNA, translated *in vivo* to provide the encoded influenza virus antigens for stimulating both T- and B-lymphocyte immune responses. Accordingly, we considered that *in vivo* induction of anti-HA and -NP specific antibodies would be possible.

However, immunization with the comparator “RepRNA + c-di-AMP” and “[RepRNA/PEI] + c-di-AMP” were overall poor at inducing detectable anti-HA or anti-NP antibody responses; any detectable IgG titers were close to non-specific background levels observed with pre-vaccination bleeds especially with the HA antigen (**Figure 7C**).

Compared to the previous groups, VRP immunization induced superior humoral responses against RepRNA-encoded influenza virus antigens, detectable in all animals (**Figure 7C**: “VRP”, anti-HA: p<0.001; anti-NP: p=0.047). Interestingly, immunization with the HA-encoding VRP induced higher titers than those observed following immunization with the NP-encoding VRP. Moreover, tight grouping of the titers from individuals was observed for anti-HA antibodies, relating also to higher titers from all individuals (titers IgG anti-HA: 8047.9 ± 597.3; anti-NP: 1014.7 ± 957.7).

It was then considered that the anti-NP response induced by the VRPs might be influenced by the influenza virus strain employed for the vaccination or analyses. In order to understand better why anti-NP IgG titers were more moderate than the anti-HA IgG titers following VRP immunization, we performed ELISA by coating with recombinant NP proteins from two different influenza virus strains: A/Brisbane/10/2007 (H3N2) and A/Thailand/1(KAN-1)/2004 (H5N1). This had no influence on the anti-NP readout compared with the anti-HA readout (data not shown).

Taken together, these results showed that VRP immunization induced strong anti-HA and anti-NP humoral responses. Overall, this confirms the requirement of RepRNA to be properly packaged for efficient translation of the GOI.

## Discussion

Several mRNA-based vaccines are currently in clinical trials to prevent COVID-19 and plan to file for emergency approval. In comparison to mRNA, self-amplifying RepRNA require very low dose and result in prolonged and increased transgene expression (5). We chose CSFV RepRNA, due to its positive-strand and non-cytopathogenic nature, thus, providing long-lasting protein expression in the targeted cell (6, 38). This contrasts with alphavirus and flavivirus replicons (11) derived from cytopathogenic viruses, often resulting in high albeit short-lived antigen expression levels; the contrast is even further from replicons based on vesicular stomatitis virus (VSV) which is both highly cytopathogenic and a negative strand virus precluding application of synthetic nanoparticulate delivery. The latter delivery has been achieved successfully using the CSFV RepRNA (6, 23–27). This formulation protects the highly RNase-sensitive RepRNA, and overcomes the obstacles for naked RepRNA to survive in biological environments and cross the cell membrane barrier to promote intracellular delivery to DCs.

Prior to such reports on delivery of RepRNA with synthetic nanoparticulate systems, delivery had been achieved using VRPs (9, 13). While we have demonstrated nanoparticle delivery of RepRNA using different formulations based on chitosan-alginate, polyplexes including PEI-polyplexes and lipids (6, 11, 23–27, 44), there is no comparison with RepRNA packaged in VRP (38, 39, 45). Another critical issue is that RepRNA delivery has been assessed primarily in mice, which may not provide accurate assessment of efficacy for humans, particularly when targeting to DCs is sought. An alternative immunological model closer to humans is the pig. The CSFV RepRNA has been delivered as VRPs to pigs (38, 39, 45). Moreover, CSFV shows at best a poor capacity to infect mice, is not applicable in humans, but is highly infectious for pigs (46).

CSFV RepRNA delivered as VRPs have been assessed in pigs as vaccines against CSFV (38, 39), wherein very efficient induction of antibody responses against the main CSFV surface glycoprotein E2, and protection against challenge virus infection were observed (39). This work did not employ RepRNA encoding foreign gene vaccine antigens. Moreover, information on T-lymphocyte activity is notably sparse, and no comparison has been made with synthetic nanoparticulate delivery, particularly of RepRNA expressing foreign gene products such as influenza virus antigens. Accordingly, we sought to assess delivery of RepRNA encoding influenza virus HA and NP by VRP *in vivo*, and comparing with PEI-polyplex delivery reported as being particularly effective (24).

Pigs immunized with VRPs strongly displayed both cellular (see **Figures 4**–**6**) and humoral responses against the RepRNA-encoded HA and NP (see **Figure 7C**). The humoral response against NP was lower than that against HA, but still notable. In our opinion, this may reflect the observations about NP epitopes being more important for inducing cellular immune defenses, while HA epitopes are certainly critical for potent humoral responses. Such readouts are considered important due to the requirement for activation of both cell-mediated and humoral arms of immune defenses to confer potent immunity against influenza.

The strong anti-HA humoral response that we observed for the VRP group is in line with results obtained with the two forms of vaccine currently in use: live attenuated influenza vaccines (LAIV) and inactivated influenza vaccines (IIV). Both LAIV and IIV induce neutralizing antibodies against the viral membrane surface glycoproteins HA and NA [for a review, see (47)]. LAIV is administered i.n. and contains the full complement of viral components, whereas IIV is administered i.m. and contains inactivated viral components, mainly purified HA antigens. In this context, our RepRNA-based strategy – employing either VRP or polyplex as delivery vehicles – relates to a combination of particular elements in the LAIV (replicating vaccine providing both HA and NP) and IIV strategies (parenteral immunization). RepRNA coding for NP are employed for inducing the cellular immune defenses crucial to provide cross-protective immune responses against influenza virus [for a review, see (48)]. Cytotoxic T cell epitopes, such as those found on the NP, are located on internal influenza virus proteins; thus it is difficult to induce anti-NP responses, because these antigens are less immunogenic than the surface proteins of HA and NA. Despite several formulation strategies to induce influenza-specific T cell responses, there is currently no T cell-based influenza vaccine on the market (49). This makes our RepRNA-based vaccine formulation advantageous, particularly with VRP-delivery, since we detected strong CD4^+^ T cell and significant CD8^+^ T cell proliferation in response to recombinant NP (see **Figures 4** and **5**).

Packaging the RepRNA in a delivery vehicle ensured the maintenance of RNA integrity *in vivo*, whether the packaging/delivery employed VRPs or synthetic nanoparticles, as in our previous reports (6, 11, 23–27, 44). Naked RepRNA is a large and labile molecule – a single cleavage would severely impair translation and abolish self-replication. From our studies, there is no other option for assisting and protecting it for efficient DC targeting. In contrast, naked RepRNA co-administered with c-di-AMP can offer some protection to the RNA; this formulation was only capable of translating in certain cases – detectable immune responses against the encoded antigens were induced in one out of five animals. This was less robust compared to the immune responses quantified in pigs receiving packaged RepRNA – VRP or polyplex.

In addition to the encoded vaccine antigens, the CSFV RepRNA encodes a leader autoprotease – N^pro^ – which abolishes type I interferon induction (50). Therefore, to promote DC maturation – and subsequent induction of adaptive immune responses – an essential requirement is the co-formulation of CSFV RepRNA with an adjuvant to activate the pathogen–associated molecular patterns (PAMPs). Hence, the choice of co-administrated c-di-AMP adjuvant, which potentiates robust humoral and cellular immune responses, including cytotoxic and multifunctional T cells – the latter have been related to robust protective T-lymphocyte immunity (30). In all likelihood, the chosen administration route and use of c-di-AMP as adjuvant might have favored the efficacy of naked RepRNA in 20% of the animals. Indeed, c-di-AMP induces a self-limited local activation of type I IFN and TNF by acting as STING agonist and then targeting it to degradation (51). Therefore, using a potent adjuvants, such as c-di-AMP, will probably help to reduce the concentration of the RepRNA packaged in PEIs due to the demonstrated dose sparing capacity (29).

Previous studies on potential influenza vaccines showed that vaccine based only on influenza virus NP proved unusable. VRP vaccines encoding influenza virus NP alone were not efficacious in pigs for protection against H1N1 and H1N2, leading even to signs of enhanced inflammation in terms of increased microscopic lung lesions, virus shedding, and increased levels of IFN-α and IL-6 (45). Moreover, one cannot rely on evaluation in the mouse models as guaranteeing success in other species, including human. NP-based vaccines have been disappointing in ferrets – considered as a good model for human influenza – contrasting with results obtained in mice (52, 53). Although NP represents an interesting vaccine antigen being well conserved across subtypes, it was insufficient to confer a complete protection to a challenge with influenza virus (54). Our formulations have the advantage of carrying an equimolar mixture of RepRNA encoding HA or NP influenza antigens. This led to specific humoral and cellular responses against both HA and NP. This is of importance, since the role of CD4^+^ T cell memory in mediating a protective immunity to influenza has gained interest the last decade [for a review, see (55)]. Moreover, pre-existing influenza-specific CD4^+^ T cells, but not CD8^+^ T cells, correlate with disease protection against pandemic H1N1 (A/CA/07/2009) in humans (56). Altogether, these findings highlight the importance to target several antigens to induce a broader immune response against influenza virus.

Our goal has been to generate RepRNA constructs as the specific active ingredient of influenza vaccines, with the potential for inducing broader and more robust immunity than the current seasonal, inactivated vaccines. Due to frequent antigenic changes of influenza viruses in the human population, current seasonal influenza vaccines must be updated annually to include the latest predicted strains. The design of a new CSFV RepRNA constructs encoding influenza virus antigen from any new isolate is a rapid and cost-effective strategy: our current RepRNA carries insertion sites that efficiently facilitate the accommodation of influenza virus genes of interest for broad protection against the virus. Technically, update of a CSFV RepRNA construct employs standard molecular biology methods to replace the GOI by an influenza virus gene (NP, HA but also neuraminidase (NA), M1 or M2) encoding antigen of the new reported isolates (57). Protection against a wide range of strains/subtypes would require a vaccine formulation mixing several RepRNA constructs carrying influenza virus genes from different strains. For the pigs employed in this study, the vaccine contained RepRNA encoding as GOI either the HA or NP from Influenza A/chicken/Yamaguchi/7/2004 (H5N1), a highly pathogenic avian influenza virus that remains a public health threat (37). This experimental approach proved to be efficient, since significant B cells, CD4^+^ and CD8^+^ T cell responses were readily detected against the RepRNA-encoded influenza virus HA and NP; moreover the increased cell number and proliferation level of the CD4^+^CD8^+^ T cell suggested the induction of a memory response (58, 59). In order to consolidate these results, the next step will be a broader formulation comprising Rep-NP and a panel of Rep-HA encoding for seasonal and pandemic influenza virus antigens (H1N1, H5N1, H7N1). The aim is to determine the level of heterosubtypic protection.

In conclusion, the present study demonstrates that immunization of large mammals with a combination of RepRNA encoding the H5N1 NP or H5N1 HA induces strong T- and B cell responses against both influenza antigens, relating to the capacity of VRPs – and to a lesser extent poyplexes – for targeting DCs for translation of the delivered RepRNA. This knowledge is being applied to facilitate exploitation of the full potential of RepRNA delivery platforms in the context of universal influenza vaccine design. The rapidity by which Rep-HA can be updated could circumvent the limitation of current vaccines that are strain-specific due to their focus on the immunodominant globular head domain of the HA (60). In our case, the encoding of complete HA protein most probably promotes the induction of antibodies reactive against both divergent head domains and conserved stalk. A sequential exposure to Rep-HA from different strains – or alternatively a vaccine formulation mixing Rep-HA harboring sequences of various strains – will increase the chance that immune responses broadly neutralize epitopes in the stalk. The use of recombinant NA as a vaccine antigen proved to have homologous and heterologous protective capacity (61). Altogether, this suggests that a RepRNA-based vaccine formulation comprising conserved Rep-NP, several Rep-HA encoding several influenza strains (seasonal plus pandemic H1N1, H5N1, H7N1), as well as the NA subunit, in combination with a potent adjuvant would facilitate the design of a universal influenza vaccine. Finally, the rapidity with which RepRNA can be modified and formulated with the delivery vehicle will be of great help to tackle emergencies, such as the Covid-19 pandemics.

## Data Availability Statement

The raw data supporting the conclusions of this article will be made available by the authors, without undue reservation.

## Ethics Statement

The study in pigs was performed in compliance with Swiss animal protection law (TSchG SR 455; TSchV SR 455.1; TVV SR 455.163) under the authorization number BE77/16. The experiments were reviewed by the cantonal committee on animal experiments of the canton of Bern, Switzerland, and approved by the cantonal veterinary authority (Amt für Landwirtschaft und Natur LANAT, Veterinärdienst VeD, Bern, Switzerland). Experiments in mice and rabbits were conducted at HZI and approved by the ethical board and conducted in accordance to the regulations of the local government of Lower Saxony, Germany (license No. 33.42502-04-13/1281).

## Author Contributions

All authors contributed important elements to the work presented in this paper. TD designed, performed, and coordinated the pig *in vivo* study, assembled the data, prepared the figures, and wrote the manuscript. TD produced the RepRNA (HA and NP) and performed the functional assays of all RepRNA batches to select the ones usable for the pig *in vivo* study. TD performed the ELISA measurements. NR designed the Rep-HA and Rep-NP and participated in the design and execution of the pig *in vivo* study. MG produced the VRP-[Rep-HA] and the VRP-[Rep-NP]. LT-H performed the mouse and rabbit experiments with the VRPs in comparison with chitosan-nanoparticle delivered RepRNA. TE, KS, and CG contributed to the design of the study, performed the mouse experiments with RepRNA delivered by various nanoparticle/lipoplexes/polyplexes, provided the c-di-AMP, and edited the manuscript. KM overviewed the design of the various experiments, discussed the results and planning, and edited the manuscript. All authors contributed to the article and approved the submitted version.

## Funding

The work was funded by the EU FP7 Project UniVax (HEALTH-F3-2013-60173) and the Swiss National Science Foundation (grant 310030_150008).

## Conflict of Interest

Application of delivery vehicles for the delivery of RepRNA vaccines to DCs (23) using replicons derived from classical swine fever virus, as employed in this paper, has been filed for patents in Europe, USA, Canada, and India, with priority date of 2008. The filing was by the authors KM and NR, together with Jon Duri Tratschin (all three as inventors) (WO 2009146867) (6), and assigned to their employer – the Institute of Virology and Immunology. This does not alter the authors’ adherence to the policies of sharing data and materials. CG and TE are named as inventors in a patent application covering the use of c-di-AMP as adjuvant (PCT/EP 2006010693).

The remaining authors declare that the research was conducted in the absence of any commercial or financial relationships that could be construed as a potential conflict of interest.
